# Cropping synonymy: varietal standardization in the United States, 1900–1970

**DOI:** 10.1007/s40656-023-00574-7

**Published:** 2023-07-12

**Authors:** Tad Brown

**Affiliations:** Department of History and Philosophy of Science, University of Cambridge, Cambridge, UK

**Keywords:** Agriculture, Standardization, Crop diversity, Intellectual property, United States

## Abstract

This article examines crop varietal standardization in the United States. Numerous committees formed in the early twentieth century to address the problem of nomenclatural rules in the horticultural and agricultural industries. Making shared reference to a varietal name proved a difficult proposition for seed-borne crops because plant conformity tended to change in the hands of different breeders. Moreover, scientific and commercial opinions diverged on the value of deviations within crop varieties. I review the function of descriptive difference in the seed trade and in the framework of evolutionary theory before examining the institutional history of varietal standardization. Pimento peppers are used to represent how vegetables were treated differently than cereals. Lack of stability within a popular pimento variety caused problems for food packers in middle Georgia, which public breeders addressed by releasing new peppers. To conclude, the article questions the role of taxonomy in intellectual property, as breeding history and yield became defining attributes for making varietal distinctions.

“Things do not really ‘exist’ until somebody writes a bulletin about them.”

Harry V. Harlan, *One Man’s Life with Barley*, 1957

## Introduction

Early in January 1930, the director of the Georgia Experiment Station, H.P. Stuckey, received a letter about the nomenclature of pimento peppers. The chief of the United Stated Department of Agriculture (USDA) Vegetable Crops Section had written Stuckey to avoid listing “two entirely different varieties under the same name” in a federal report.^1^ The term “pimiento” is Spanish for pepper, but its American slack-jaw equivalent “pimento” refers specifically to a group of thick-walled sweet peppers.^2^ A breeder in California had developed a globular pimento variety with seed sent in 1911 by an American consul in Spain. Its trade name was simply “Pimento.” From the same imported Spanish seedstock, S.D. Riegel & Sons in Experiment, Georgia (also home of the Georgia Experiment Station) bred a cone-shaped pimento called “Perfection.” The two peppers were exceedingly similar in all but form: One had four lobes, the other three, with an “entire absence of pungency” in either.^3^ The question posed to Stuckey, as to which listing took precedence, is indicative of a set of larger concerns for science, industry and law in the twentieth century.

This article is an attempt to understand how agricultural experts addressed the determination of crop varieties in the United States. Scientists tend to refer to crop varieties as a grouping ‘below the lowest taxon’, the lowest taxon being the species rank.^4^ Reflecting this status, different groups—scientists, seedsmen, and farmers—refer to the same variety by different names based on different sets of descriptive criteria, including use-value (Berlin et al., 1966; Cleveland & Soleri, 2007). The challenges with making distinctions below the species rank, in this sense, anticipate debates within the larger field of systematics as to whether organisms should be grouped by genetic relatedness or phenotypic traits, with separate outcomes possibly resulting from either (Velasco, 2008).

The question of crop classification has been notably absent in the historiography of agricultural science. Recent work on the subject has highlighted how botanical taxonomy has interfaced with social and legal concerns. For example, Helen Anne Curry (2021) demonstrated that maize (corn) taxonomy in the twentieth century drew from prevailing racialized ideas about Indigenous people in Mexico. In another case, Joceyln Bosse (2020) discussed the classification of *Cannabis* in contests over criminal possession of marijuana to show that species distinctions failed as a legal defense, regardless of botanical accuracy. This article returns to a preliminary scientific chore of taxonomic work. Grouping crop varieties depends on being able to identify them. Molecular analyses have become prominent for determining varietal identity in the laboratory, but not to the exclusion of older techniques (Holmes, 2017). Regardless of the chosen method, decisions about how to tell one plant variety from another depend on who has the authority to officiate between them and the criteria employed in doing so.

**Fig. 1:**
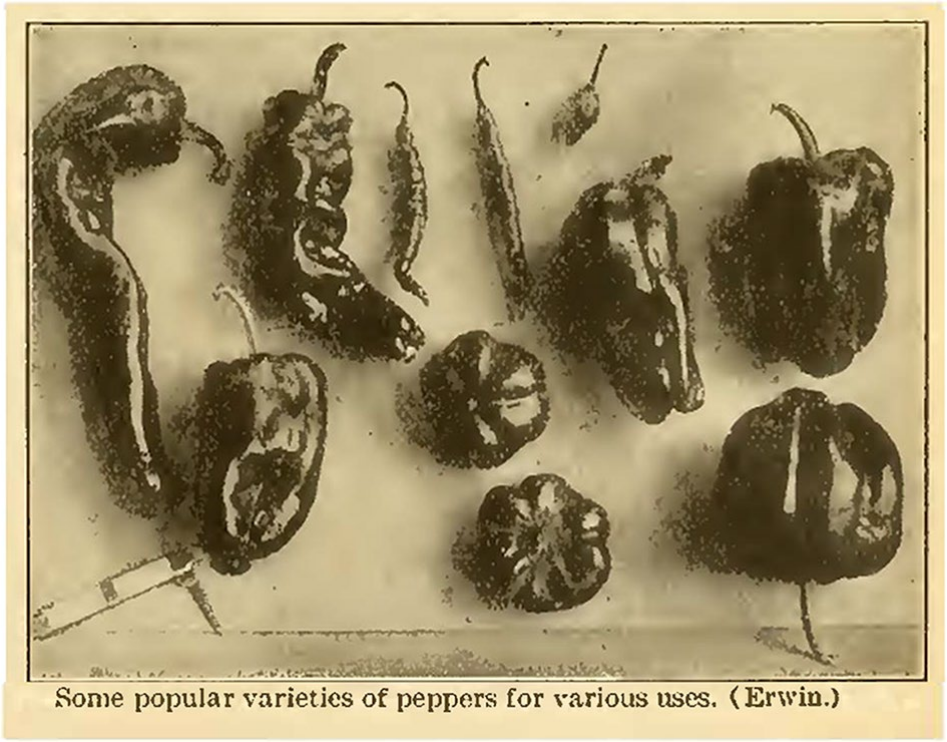
Image of pepper varieties from ‘Vegetable Garden and Trucking Crops’ in *Practical Farming and Gardening.* Reproduced from Erwin (1914), p. 105

Specialized expertise is what prompted the chief of the USDA Vegetable Crops Section to send his query to Experiment, Georgia. According to Stuckey, Georgia was “the only Experiment Station that [had] done extensive work with the Perfection Pimento” because other stations regarded it “as a miscellaneous truck crop.”^5^ Further discussion with the USDA chief revealed that the federal vegetable project was based upon organizing crop varieties by species classification. With pimento peppers, all varieties belong to *Capsicum annuum*, “the most widely known” of the five species in which peppers have been domesticated.^6^ (Other pepper varieties in the same species include cayenne, peperoncini, Anaheim, Italian Sweet, jalapeño, poblano, serrano, banana peppers, and thousands more.) Thus, this particular question came down to which trade name—Perfection or Pimento—to publish on the list as representative of the type (Fig. 1).^7^

For his own part, Stuckey always recommended Perfection to his constituents for commercial growing.^8^ The choice was a matter of regional pride. A significant industry emerged in middle Georgia on the merits of Perfection. In fact, a processing plant in the vicinity of Experiment—founded in part by Riegel—led the industry in packing fire-roasted pimento peppers (Cochran, 1951; Brackett, 2018). Minced and mixed with cheese and mayonnaise, “pimento cheese” became a popular sandwich spread throughout the region (Wallace, 2010). Typical of the times, six cans of pimento were listed as emergency items in *A Thousand Ways to Please a Husband with Bettina’s Best Recipes,* dedicated to “her whose ‘Bob’ is prone to wear a sad and hungry look/ Because the maid he thought so fair/ Is—well—she just can’t cook!” (Weaver & LeCron, 1917, p. 18). By 1930, Georgia furnished half of the pimento peppers consumed in the United States, having outpaced California as the nation’s largest producer (Brackett, 2018).

**Fig. 2:**
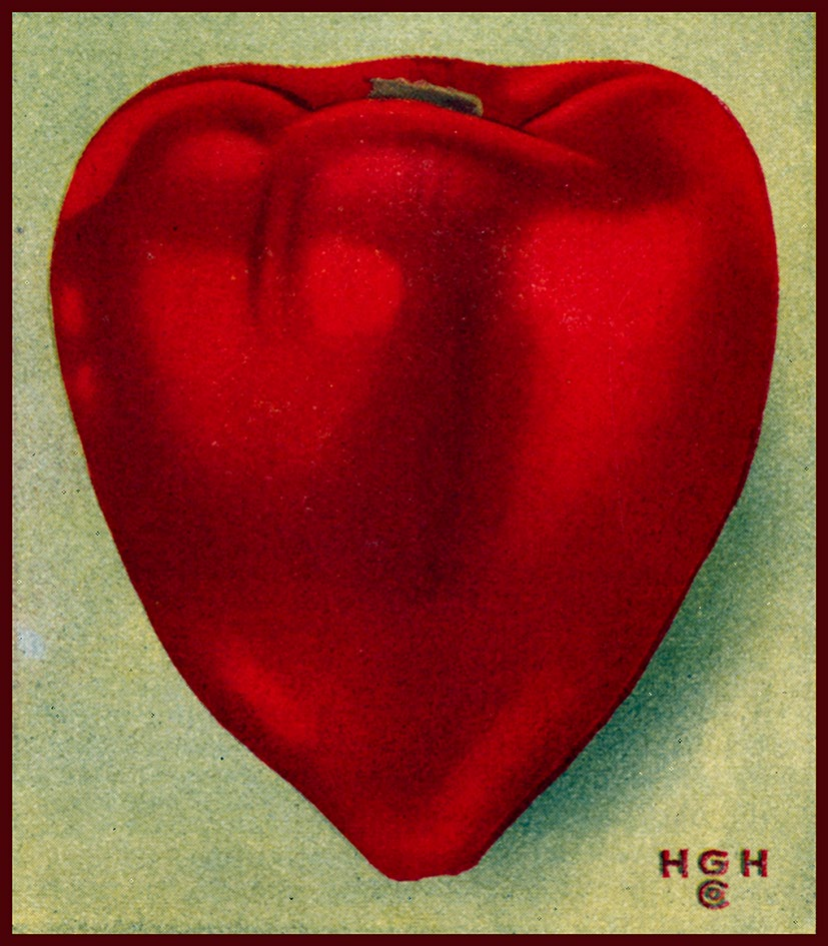
Seed catalogue image of Hastings’ Perfection Pimiento. Hastings’ *Seeds* (Spring 1927) Atlanta: H.G. Hastings Co. Credit: Kenan Research Center at the Atlanta History Center

Out of mutual interest, Stuckey forwarded the question about pimentos to H.G. Hastings, owner of Hastings’ Seeds in Atlanta, Georgia.^9^ The seed company distributed a mail-order catalogue each spring and fall, offering a choice of flowers, field crops, and vegetables, which included a section titled Hastings’ Georgia Peppers. Among the mixed offering of hot and sweet peppers was the variety “Hastings’ Perfection Pimiento.”^10^ What Hastings offered, since as early as 1925, was a version of Perfection. The seedman had noticed variation within the pepper and, like Reigel before him, selected it to his own standards. For doing so, Hastings exercised the prerogative to sell his stock under an amended name. Depending on the retailer, the public could choose between Pimento, Perfection, or Hastings’ Perfection Pimiento. Without agreed-upon criteria for making varietal distinctions, who could say whether Hastings’ pimento warranted an eponymous prefix, or if Perfection itself was nothing more than a regional alias of Pimento (see Fig. 2).

A correspondence ensued between Hastings and the chief of the Vegetable Crops Section in which the proprietor sympathized with the USDA’s desire for accuracy. After all, Hastings had served as the president of the American Seed Trade Association in 1920. He nonetheless felt obliged to admit that “there is a sort of twilight zone where it is very difficult to determine what is new or what is distinct sufficient to justify a new name, and what is not.”^11^ What is more, based on his own experience, Hastings noted that when it came to nomenclature the public showed “relatively little respect for the viewpoint of the scientist or to what the seedsman might prefer.”^12^ The chief, in his response to Hastings, underscored that seed trade catalogues were complicit in producing knowledge about the relatedness of plants.^13^ He had as a child been introduced to plants this way, and argued that formatting commercial content by scientific classification could help dispel public misunderstandings. As it was, seed retailers had an objectionable habit of listing plants of separate species under the same section in promotional material.  

Cataloguing in the seed industry was different from the USDA approach of listing varieties by species. Species assignments, as defined by an ability to reproduce fertile offspring, were subject to empirical review. Field experiments provided a method for falsifying whether or not a variety belonged among other varieties as conspecifics within the same species. Seed retailers formatted their offerings to generate sales, at times dispensing with taxonomy. The grievances with the seed trade did not end with species, yet recourse to science faltered below this terminal rank. The “twilight zone” of varietal identity lacked an unequivocal proof of membership. Whether a pimento pepper plus-or-minus one lobe classified as an entirely different variety was a matter of opinion. The history of varietal standardization in the early twentieth century offers perspective on the scientific criteria employed to delineate between named crop varieties.

In what follows, the category of variety is placed within the larger history of taxonomic botany and agricultural science. I review early attempts to understand and address the problems of plant identification. The next section introduces the Committee of Varietal Standardization and provides an account of its historical origins, founding members, and stated ambitions.^14^ The three subsequent sections explain the technical protocols employed by the Committee: classification, testing, and registration. As I show, varietal standardization was achieved by subsuming the ambiguous biological realism of “variety” to that of yield. The article then shifts away from the registration of cereals and back to vegetables to explain the treatment of different crops. With this, the discussion returns to pimentos to illustrate how varietal instability affected the food processing industry. It concludes by reflecting on how the administrative history of science relates to the history of intellectual property in plants.

### Singling out something of the sort

Synonymy, or multiple names being applied to the same kind of organism, was a longstanding problem in taxonomy. Colonial expansion and new methods of scientific field survey exacerbated cases of redundancy, as the description of unfamiliar flora produced a wealth of new information about plants (Kohler, 2013).^15^ Repeat discovery bloated the annals of science, especially as elite European institutions lost undisputed command over taxonomy to American experts, who fought to christen New World flora with self-important titles (Kingsland, 2005). Negotiations over whether botanical material was sufficiently dissimilar to warrant a new taxon bogged down the ever-expanding description of the plant kingdom. Synonymy was one problem. The field of plant taxonomy also suffered from the inverse scenario known as homonymy, where one name mistakenly refers to separate taxa. Either way, imperial botanical gardens spearheaded the movement to assign a single name per species to facilitate the sharing of new materials and clarify correspondences about the natural world (Müller-Wille, 2001).

Historians of science have illustrated how the Linnaean binomial achieved worldwide success, redefining who had the privilege to bestow a plant with its official name (Kingsland, 2005; Kevles, 2007; Müller-Wille, 2003). In time, botanists adopted the practice of designating a type specimen for each taxon—an actual physical example of plant, dried and archived—to be referred to by its first published name. One name, one sample, one taxon. Lorraine Daston (2004) dubs this act “words made flesh by lottery” because, as the point of reference, the type specimen was arbitrary in all but priority, compressing an abstract class into one almighty candidate. If with type specimens, one object came to stand for the existing forms a species could exhibit, something else occurred for crop varieties. Deviations within a single variety became prospects for originating new named varieties. Rather than subsume difference within a single kind, crop variation was either to be encouraged, as that from which new kinds emerge, or eliminated.

The proposition of a stable crop variety was long perceived as an oxymoron, in that any trait difference had marked a divergent species (Müller-Wille & Orel, 2007; Ratcliffe, 2007). That Darwin equivocated on the distinction between species and varieties is telling (Hamilton, 2014). By refusing to reify the difference in rank, breeders’ first-hand accounts helped Darwin make sense of selection as a mechanism for speciation.^16^ The Darwinian research program put the practical breeding of stockmen and gardeners within a framework of natural history (Varno, 2011). The mechanism responsible for populating the earth with wild biological diversity shared a parity with selective breeding, yet the deep chronologies of speciation averted certain issues confronting lineage histories on the farm. The theory of descent with modification did not resolve when the generations of genealogy capitulated to the types of taxonomy.

With the turn of the twentieth century, the genotype–phenotype distinction began to rework how biological differences were conceived. Historians now widely accept that the genetic theory of inheritance did little to change plant breeders’ practices before 1930.^17^ The variety remained the organizing unit of trade as well as the primary product of agricultural research. For instance, Staffan Müller-Wille (2005) illustrated how, at the Svälof Breeding Institute in Sweden, the need to release new varieties obstructed the study of heredity, denying research and record-keeping that was designed to “break the type.” What Mendelism did was reorganize the institutions involved in breeding, the flows of funding, and the style of reasoning (Bonneuil, 2006). It also helped shift the responsibility for seed that did not germinate true-to-type from the breeder to the merchant supplier (Radick, 2013). As such, the notion of a reputable commercial variety became synonymous with a fixed type.

According to Dominic Berry (2014), British plant breeders continued to rename existing varieties after World War I, despite oversight by the National Institute of Agricultural Botany. “Rather than merely seeing synonyms as evidence of fraud,” as has been typical, Berry frames synonymy as evidence of another historical kind: That the popular scientific rhetoric of pure-line theory failed to withstand material scrutiny (2014, p. 27). Put simply, varieties remained instable. State officials in Great Britain lacked the bureaucratic authority to refute claims of varietal distinctness, even with a sizable public research infrastructure devoted to agricultural genetics (Charnley, 2013a). Seed firms, at odds with government opinion, backed their claims to an amended varietal name by citing personal history with breeding lines. In other words, Hastings’ action—of identifying, selecting, multiplying, and naming a variety—was ordinary practice, given that varieties were liable to change.

In the United States, more so than Europe, the science of genetics afforded a class distinction between agronomists and the rest of the farming public. Experts in America barred amateurs entry into the scientific pursuit of crop improvement (Harwood, 2015; Palladino, 1994). The reason for this stance can be discerned from the nature and funding of state institutions. In her review of the American Breeders’ Association, Barbara Kimmelman (1987) argued that public breeders were most willing to entertain ideas about Mendelism by dint of its ties to evolutionary theory. Careers advanced on the touted prospects of Mendelian genetics.^18^ The study of hybridization—construed as an exercise in the predictability of scientific laws—reinforced this self-perception of agricultural experts as experimental scientists (Kimmelman, 1983, p. 167). While results varied, commitment to crossbreeding, as a method, depended on pairing distinct strains, varieties, or species. The research agenda of hybrid breeding put emphasis on the difference between kinds, as did the marketplace for plants.  

### Reporting vegetal properties

By the turn of the century, scientists had convened for decades to set international nomenclature rules to end synonymy in botany. Episodes of ill communication in the horticultural trade prompted similar calls for action in the United States.^19^ Organizers rallied around the prospect of “making buying easier” by enacting measures to ensure that people got what they expected when purchasing plants. Experts had long recognized that variation within named types posed a significant commercial problem. Dating back to the previous century, orchardists and nurseryman led the push for varietal standardization (Kevles, 2011; Pawley, 2016). This precedent convinced scientists that progress in the commercial vegetable market depended on a concerted initiative by the seed trade.

Questions of varietal naming were complicated by ongoing concerns about the consistency of varietal identification. In January 1906, at its second annual meeting, the American Breeders’ Association appointed a Committee on Breeding Vegetables. Its chairman delivered “a hurried survey” the following year and stated that vegetable breeding would benefit from efforts “to fix the types we already have and … to produce new forms”, with a specific mention about shortcomings in the current offerings of cabbages and tomatoes (Emerson, 1907, p. 265). The chairman also recommended an inventory of existing types to capture the state of horticultural art, highlighting the need to establish standards for making judgments between types. Without such an approach, the most exhaustive catalogue of vegetable varieties would amount to little more than so many names.

At the same meeting, committee member W.W. Tracy, who worked for the USDA, submitted a paper titled “Importance of Full and Accurate Descriptions of Varietal Differences in Growing Seed of Market Vegetables.” Presenting before the entire association, he too lamented the lack of “clear, definite, and explicit descriptions of just what any particular variety should be” (Tracy, 1907, p. 199). Tracy believed that an exact testimonial for each variety would aid vegetable breeders in reproducing seed that retained its ideal characteristics. The following year—now with Tracy as chairman—the Committee on Vegetable Breeding motioned to submit vegetable varieties for methodological testing in different environments. As it stood, Tracy decried “a want of uniformity” in naming practices and a “want of knowledge” about the variation within different stocks of the same variety (Tracy, 1908, p. 233). Only through systematic review could each distinct varietal name obtain a full and accurate description.

Proposals for an assessment of vegetable varieties carried an aspirational quality. The goal in all of this was to encourage breeders to quit breeding for the sake of useless variation and, instead, devote more effort to the development of uniform stocks with desirable characteristics. For professionals concerned with the reliability of commercial seed stocks and the overall state of agriculture in the United States, simplifying the diversity within any single vegetable variety was an important component of making comparisons—between institutions, field plots, and seed samples. The seed certification movement in the United States shared similar concerns about the quality and productivity of commercial seedstocks (Cooke, 2002). An established reference for each named variety conveyed the possibility for scientists to clearly communicate research findings and direct collective efforts.

The American Breeders’ Association was one among multiple institutions engaged in the description of vegetable varieties at the turn of the century. At the 1904 annual meeting of the Society for Horticultural Science, Lee Corbett of the USDA articulated a plan for conducting uniform tests on crop varieties. He noted that trade descriptions were devoid of “any fixed notion regarding varieties”,—other than to attract greater sales—when what scientists desired was a standardized format for indexing varieties, the object being a “type description” (Corbett, 1905, p. 69). In one attempt to ascertain the extent of variation within crop varieties, the Pennsylvania State College and Experiment Station conducted a four-year study on cabbages and tomatoes. The findings demonstrated that seed of the same variety exhibited marked differences, leading the investigators to conclude that “improper breeding” had produced a number of strains within each variety (Myers, 1912, p. 86) Aside from discrepant pedigrees, experiments across the United States also illustrated that plant varieties adjusted to new environments, especially soil types (Burgess, 1912). An unfavorable habitat could discredit a named variety, when the same stock evaluated in other settings may be deemed superlative. Scientists needed standards to control for these confounding vagaries.

In 1915, a committee was formed within the Society for Horticultural Science to devise a workable scheme of varietal standardization. The proposal sought to delimit how scientists recorded observations on the nature of crop varieties’ conformation and performance. None other than Tracy served as the chairman for its Committee on Score Cards for Vegetables (Tracy, 1916). Tracy endorsed the score card as a format for furnishing an almost-mechanical description of each plant part. The rubric would aid in varietal comparison and help reset the priorities for how to judge superlative crops (c.f. Fitzgerald, 1990). Unfortunately, as it stood, few systematic trials existed for the category of vegetable varieties. There was the early investigation by Tracy on lettuce varieties (1904) and a similar report on American bean varieties authored by Chester Jarvis (1908). A decade later, a careful study of potatoes by William Stuart (1918) emerged as the leading example of how best to proceed. These publications captured the state of varietal diversity for their respective crops through type descriptions.

Not all varieties submitted readily to standard descriptions. According to Corbett, potatoes belonged to the class of “stable or fixed varieties” that relied on vegetative propagation. The other class of varieties in his typology—those being reproduced by seed—consisted of “fluctuating or plastic varieties.” Corbett put forth a working definition of a variety that would help secure the integrity of a given varietal name by collapsing the two classes. In his verbiage, a variety consisted of a population of indistinguishable individuals. Corbett basically asked that breeders deprive seed-borne varieties of flux.^20^ What was the value of varietal tests for an unreliable make-up of seedstocks? As it stood, the commercial seed trade rarely dealt with pure strains and, as such, testing amounted to a comparison of “one mixture with another mixture” (Corbett, 1917, p. 82). Crop uniformity offered a key for standardizing the reference to named varieties.

## The Committee of Varietal Standardization

Similar problems of conformity confronted the greater horticultural industry. In 1915, the American Joint Committee on Horticultural Nomenclature formed to expunge misnomers, synonyms, and “name chaos” from the market (Olmstead et al. 1923, p. xii). The organization—an upstart conglomeration of nurserymen, landscape architects, park superintendents, florists and ornamental growers—agreed that the most practical egress would be to recommend a standard name for each plant. Inspired by the avant-garde activities of the American Rose Society and the American Pomological Society, which had imposed grammatical rules on trade names to reduce duplicates, the Joint Committee inserted an asterisk beside one name per plant from the Bailey Standard Cyclopedia of Horticulture. The alphabetical index became known as the “1917 Official Code of Standardized Plant Names”. Adherence was voluntary, yet the publication enjoyed widespread authority (MacFarland, 1920). The horticulture trade and nursery industry had achieved an agreed-upon reference for the names of commercial plants.

Crop nomenclature was not included in the “Official Code.”^21^ This omission indicated a problem for the scientific community. In 1920, the chairman of the Joint Committee presented before the American Seed Trade Association to rally support for the errata of agricultural varieties. He confessed a reluctance to engage with crop nomenclature and for good reason, reciting the common impression that “a thousand vegetables are using ten thousand names” (MacFarland, 1920, p. 66). The owner of Hastings’ Seeds was in the audience and responded to the roughshod synopsis with one exacting question: “How do you arrive at what is a variety?” The chairman of the Joint Committee refused to attempt an answer, directing Hastings and his fellow seedsmen to “buy a broom and sweep your own corners” (ibid, p. 67). The broom for such housekeeping was in the hands of the Vegetable Growers’ Association of America.

At the same meeting, Francis Stokes stood before the American Seed Trade Association to introduce a plan of action by the Vegetable Growers’ Association. He was speaking on behalf of the organization of vegetable growers but was also an active member of the seed trade. To summarize, the scheme he put forward included the adoption of a definite code of nomenclature, a list of existing vegetable varieties, and an official accrediting body for registering new varieties. Fully aware of the personalities present, Stokes expected “a great many members to take exceptions, to rise and cry, ‘impossible,’ and to damn the whole principle” (ASTA, 1920, p. 54). Seed firms and mailing houses of the United States had enjoyed the liberty of self-governance for decades. There was a strong sense that taxonomic imperatives could be established within the ranks and monographs of scientific botany, but exporting the exercise into the horticulture trade conflicted with breeders’ practices.

Stokes’ disclaimer was prescient. Certain members of the seed trade responded to the resolution with immediate disapproval. One dissenting voice summarized the scheme as an “attempt to deprive people, individuals, of the right to exercise their individual discretion in naming or renaming or re-renaming or re–re-renaming” their goods (ASTA, 1920, p. 56). Discussions about standardization in the seed trade often returned to reconciling the viewpoints of buyers and sellers. Merchants expressed consensus on two general opinions. Firstly, that market demand should determine the elimination of varietal listings and not the priority of name; for as had been known to happen, seed firms, knowingly or unknowingly, introduced identical seed stocks under different names. Often a catchy name outsold the same seed originally marketed under a nondescript moniker. Secondly, any bid to make varietal offerings around the country exactly the same would be, in the words of one seedsman, “a detriment rather than a benefit” (ASTA, 1915, p. 90). Different growing conditions asked for modifications in type, and it was their commercial duty to deliver varieties fit for each market.

Just as importantly, recent history hinted at the futility of the enterprise. Similar motions had been suggested for years, always lapsing without effect. The president of the American Seed Trade Association, H.G. Hastings, had chaired a committee tasked with the objective of cropping synonymy. Now defunct, the committee found it “a stupendous undertaking” and one without direction (ASTA, 1920, p. 58). Hastings recalled how he had met with “Experiment Station and College people” for the express purpose of establishing nomenclature rules that would settle any classificatory questions, acting “as a court of reference in case of controversies” about varietal naming. What they really wanted, he learned, was “not so much a straightening out of the nomenclature as it was an absolute standardization of varietal types.”^22^ Hastings viewed the insistence as anything but practicable, as differences were bound to arise according to the ideas and selections of different breeders. In his opinion, only by limiting seed to a single source would a variety conform to a single name.

Hastings recognized the need for formal standards but had long doubted whether a nomenclature committee could fix varietal identity. Rules for the naming of crop plants depended on adopting a code of grammatical principles and a system for establishing eligibility so that each article of record possessed a single designation (Ball & Clark, 1918). In this sense, the clonal articles sold by florists and nurserymen were materially different than vegetable seed. Those precedents were misleading for the class of “fluctuating varieties” sold by seedsmen because, with the latter, a name could persist without material correspondence to the variety’s initial description. A way forward, affirmed Hastings, was not to “upset the present trade variety names.” but rather to lay a foundation for registering future varieties (ASTA, 1920, p. 60). The American Seed Trade Association ultimately approved a resolution to work with the Vegetable Growers’ Association. A five-member committee was appointed, with Stokes as chairman, along with matching funds for the clerical load ahead.

Given the number of institutions with overlapping interest in the topic, what body would be responsible for adjudicating named crop varietals? Hastings deferred to a concurrent inquiry by the American Society of Agronomy, an independent scientific body founded in 1907 by USDA officials and members of land-grant agricultural colleges. At its 1919 annual meeting, the president of the American Society of Agronomy appointed a ten-member Committee of Varietal Standardization (hereafter, the Committee) to survey crop varietals and suggest a standard name for each (Oakley, 1920). While the Committee chairman R. A. Oakley worked for the USDA Bureau of Plant Industry, six of the other eight cohort members were employed by agricultural experiment stations. H.G. Hastings—peddler of his own Perfection—was the only Committee member not working directly for the government.

### Classification: varieties upon varieties

To begin in earnest, the Committee needed to adopt a definition of a “variety”. The meaning of this term would guide crop specialists in making their determinations. Up until 1923, the Committee conducted its business through personal correspondences. After back-and-forth debate, a variety became defined as “any group of plants morphologically indistinguishable from one another” but readily distinguishable from other such groupings within the same species. (A “strain” entailed the same definition but within a variety.) Only half of the Committee supported the elected definition (Agronomic Affairs, 1923a, b). Among the dissenters, this notion that varieties could be discerned “based upon morphological characters alone” showed too little regard for the fact that types could be identical in a taxonomic sense yet diverge in productivity (Agronomic Affairs, 1925). What mattered for agriculture was productivity.

As Deborah Fitzgerald (2008) illustrated, an industrial ideal came to dominate American agriculture in the 1920s, remodeling food production on the business practices of the factory. Uncertain botanical identity was incompatible with the measurable demands of industrial progress. Indeed, the uniform variety became the widget by which the machinery of a modernized agriculture would operate. Systematic large-scale breeding experiments and standardized record-keeping achieved what Christophe Bonneuil (2016) has called “industrial simulacra” in crop plants. Variation was effectively bred out of varieties. 

Members of the newly-minted Committee were already involved in cataloguing the crop diversity available on the American landscape. The Committee delegated each crop, in bureaucratic fashion, to an appointed subcommittee of qualified experts. In addition to investigations by Allen Clark on wheat (Clark et al. 1926a, b) were those by Harry Harlan for barley (1926) and T.R. Stanton with oats (Stanton et al., 1926). The Committee’s reluctant definition soon revealed its real-world limitations. Stanton observed that any attempt to “differentiate these numerous strains [of oats] by the use of minute, obscure and otherwise unsatisfactory, morphological characters,” was “not practicable” (Stanton et al., 1926). These investigators confirmed that the classificatory impulse of natural history fit awkwardly with the aims of agriculture.

Defining varieties based on appearance did not translate into crop selection. In Harlan’s opinion, the big challenge was in distinguishing those useful physiological traits from those attributable to place, though he deemed both “worthless as taxonomic features.” For example, Harlan arrived at the “density” of grain heads as the most important and overlooked character in barley. Varieties differed in density that “showed no other differences” (Harlan, 1914, p. 134). For this reason, the Committee would need to address the shortcomings of taxonomic morphology to achieve crop varietal standardization for breeding purposes. It did so through varietal testing.

### Testing: the key to the countryside

The study of agriculture, with its overt economic devotion to the harvest, operated according to a different precept than the larger field of botany. Questions of varietal classification were nevertheless inseparable from larger scientific concerns. Many of the Committee members who were responsible for deciding how to determine varietal identity were already deeply involved in field research that sought to improve crop production through applied genetics. These agronomists published in experiment station bulletins and in *The Journal of Heredity*, using breeding experiments to pursue lines of inquiry at the forefront of evolutionary science. There were notable finds, too.

Decades of scientific work with crop breeding trials provided insights into debates about evolution and species formation. The production of fertile true-breeding hybrids was an explicit goal of scientific breeding and a subject of some excitement because of its amendment to the general principles of Darwinism. A committee member from the Washington Experiment Station spent seven years making a reciprocal cross between wheat and rye species (Gaines & Stevenson, 1922). From the same breeding plots, scientists produced theoretical inferences and very practical material outputs for crop improvement programs. Committee member A.B. Conner from the Texas Experiment Station, for instance, published on “the question of whether inbreeding grain sorghum results in a reduction in vigor and a decrease in yield,” similar to findings with corn (Conner & Karper, 1924). These crop studies made important contributions yet differed from the general agenda of biological sciences in terms of the eventual clientele involved.

Economic imperatives privileged the standardization of cereals. Farmers and the national economy both stood to benefit from research on cereal synonymy because of the per-acre monetary value of grain monocultures. While fields planted in multiple varieties would not necessarily affect the crop’s market grade, the sowing of instable or admixed stocks influenced the average harvest yield (Stewart, 1919). This factor explains why the initial reports on varietal standardization focused on the American West, where crop scientists had been conducting surveys among grain farmers to determine such things as the geography of crop distribution. Committee members looked to these existing field studies to establish an empirical basis for varietal identity.

A foundational bulletin on cereal standardization came from George Stewart, a future member of the Committee. Stewart noticed that common grain varieties in Utah went by various names in the same locale. Because of this, he “believed that the first step toward successful standardization of varieties [was] to learn and apply uniformly the correct varietal names” (Stewart, 1920, p. 21). Experts repeatedly invoked the need to inventory extant varieties for classification purposes, but even then, the taxonomy of cultivated plants did not reflect actual relatedness (Ethridge, 1916). Reliance on name alone, therefore, was unlikely to produce an accurate archive of varietal diversity.

**Fig. 3:**
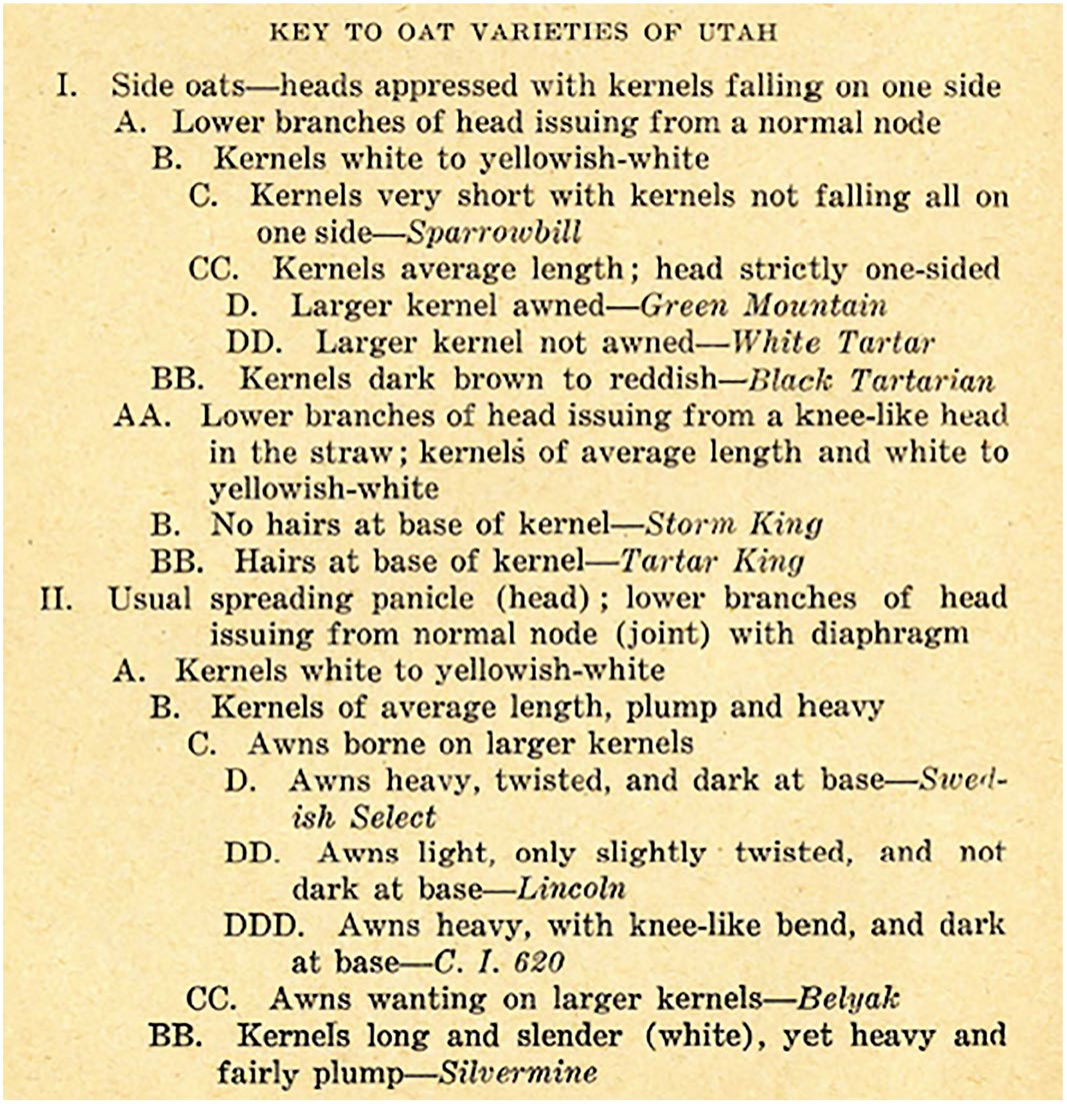
Example of varietal key. Reproduced from Stewart (1920)

To proof each varietal name, Stewart employed a stepwise key that posed binary choices between plant features, a version of the score card. Assessment of crop diversity would be done this way, by describing the most important commercial varieties trait by trait. The presence or absence of specific plant characteristics furnished fieldworkers with a systematic summary of popular crop types (see Fig. 3). By controlling for the environment, crop scientists could abstract a consistent description from varietal keys. To do this, the experimental research relied on the vast geographic spread at the disposal of state experiment stations.

Scientists aimed to test varietal morphology against changes in growing condition. Before joining the Committee, Allen Clark had enlisted the help of eighteen experiment stations for a six-year study of wheat (Clark et al., 1922). What handicapped prior studies on varietal classification, in Clark’s opinion, was the unknown influence of the environment on character expression. “The same variety often was represented by different lots of seed obtained from different sources,” and such bulk seed caused discrepancies in reporting (Clark et al., 1922). Even commercial seed could express different traits depending on pedigree and the selection pressures of diverse environments.

For Clark’s study, seed of the same variety was assigned an accession number based on its exact source, enabling a comparison of identical material across participating stations. Each study-site amassed cumulative data from “classification nurseries,” or short five-foot rows. These test plots functioned as experimental controls for determining the overall profile of a variety. Findings from the study led by Clark amounted to a massive folk taxonomic deflation for American wheat. “There were 229 varieties keyed and described as distinct varieties, although they were known by more than 800 names” (Clark et al., 1922). To be considered a valid name, the crop variety needed to withstand comparison against findings of the same variety in distant scientific trials. Few withstood the test.

What is most notable about this work is that testing for varietal identity was done in conjunction with hybridization experiments. One explicit purpose of annual field testing for the study of inheritance was to treat prospective varieties as possible parent-stock for hybrid breeding. For example, the Department of Plant Breeding at Cornell University, in cooperation with the USDA Office of Cereal Investigations, published results from early varietal tests in the 1910s. These studies conducted at the grounds of the New York Experiment Station sought “to determine the amount and nature of variation” within breeding stocks and to discover the effects of the environment on changes in varietal characteristics (Love & Craig, 1918). Cornell arranged a cooperative exchange with the Montana Experiment Station and the Agronomy Department at the University of Missouri to affirm is findings. Statistical analysis from the comparative trials of cereals (wheat, oats, barley, rye) resulted in a large data set: The information reported on a range of factors for popular varieties, new introductions, wild progenitors, and hybrids (Love & Craig, 1918). Agronomists saved any cross from the study with commercial promise.

This line of research followed on earlier investigations with corn by future members of the Committee of Varietal Standardization. As early as 1912, H. H. Love reported that the Department of Plant Breeding at Cornell University had been studying the correlation of visible characters with “yield or earliness” in seed corn (Love, 1912, p. 330). Three years of testing justified the tentative conclusion that breeding could quicken maturity and maintain seed size. Other members of the Committee also experimented with corn breeding earlier in their careers, including Herbert K. Hayes at the Minnesota Experiment Station (Hayes, 1912). Corn, an outcrossing grain having conspicuous male and female parts within reach, has a special reproductive biology. Jack Kloppenburg (2005, p. 123) summed it nicely, stating, “in no other crop is the yellow brick road of hybridization as easily traveled.” Breakthroughs with corn showcased the economic potential of hybrid seed (Fitzgerald, 1990). There is, however, a Midwestern tendency in the historiography of plant breeding in the United States to generalize from corn, when other crops found the road less easily traveled.

### Registration: please deposit your identity upon entry

Committee members were knowledgeable of the foregoing varietal trials, if not directly involved, and they were aware of certain problems attending to the field methods. This is why the Committee recommended an official system of crop registration to validate and record claims of varietal distinctness. (Members of the seed trade won the concession of grandfathering older varietal names, due to the same varieties being known by different names in different parts of the country.) Entrance of varieties within the registry entailed a number of steps. Applicants were required to submit the description of a variety, including its history, with accompanying performance records from experimental trials. This included: three to five years of comparative data, a sample of “not less than six heads and one ounce of seed,” a permissible name, and a statement of origin (Agronomic Affairs, 1922). A revolving three-person group oversaw and approved the submissions for each crop.

The scope of registration targeted qualities useful for agriculture. For this reason, any confusion over botanical identity became clarified by redressing questions of taxonomy with field data. Members held that a “variety must have a performance record significantly better than the standard commercial variety with which it has been compared” to be eligible for registration (Agronomic Affairs, 1922). The decision to enter economic considerations into the review process would have lasting importance on how crop specialists identified, described, and reviewed subject matter. Basing varietal distinctions on marketable qualities—rather than morphological features—helped stabilize the crop name for trade purposes (e.g. Hahn, 2011). Incidentally, public institutions became obligatory passage-points for most registered varieties because state and federal experiment stations generated the trial data for each application.

Members may have disagreed on the definition of a variety, but academic debate would not obstruct the shared pursuit of an industrial agriculture. Again, the stated goal of registration was couched within the breeding agenda of hybridization. The Committee made it clear that a variety must have some economic character “for the purpose of combining in one variety this favorable character with other important characters” (Agronomic Affairs, 1922). Registration was ultimately a prequalification of parental breeding stock. That is, a variety gained institutional legitimacy for what it could become in the breeding of new varieties.

No physical infrastructure existed to handle the work involved with registration. In 1923, the American Society of Agronomy signed a memorandum of understanding with the USDA Bureau of Plant Industry to curate materials of the registry within the Office of Cereal Investigations. In addition to stewarding the registry for cereals and testing new crops at its experimental farms, the Bureau of Plant Industry began to seize and prosecute mislabeled seed with passage of the Federal Seed Act in 1926. Printed declarations of varietal identity were suddenly subject to laboratory review in Washington. Most prosecutions under the law, which was limited to interstate commerce, resulted from the sale of low quality or admixed seed stocks. The issue of truth-in-labeling was more about poor germination than varietal fraud.^23^ Regardless, the increasing exercise of federal oversight indicated a perceived need for the positive identification of named varieties.

Once keyed and tested, the popular commercial cereal varieties were submitted for registration. In 1926, Clark, Stanton and Harlan presented their survey results for the three major crops to the Committee for approval. That first year, the American Society of Agronomy registered all two hundred-plus cereal varieties identified by the subcommittees, save one. Official records include no details about the rejected barley submission, but the overwhelming rate of acceptance signaled a trust in the mechanics of varietal registration. A variety became any seedstock that outproduced a comparative variety in field trials.

**Fig. 4:**
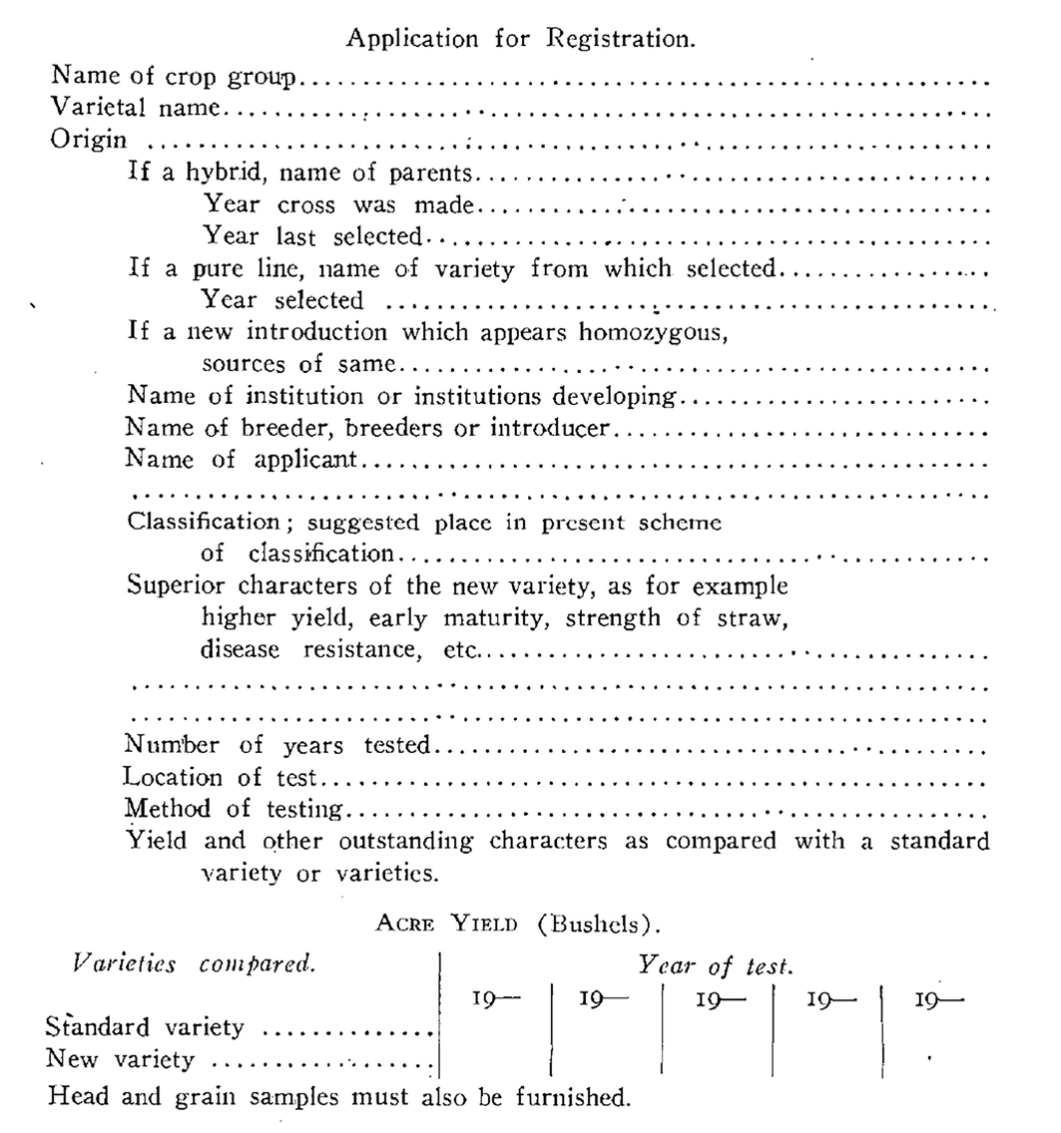
Application for Registration of Oats, Barley, and Wheat. Reproduced from* Journal of the American Society of Agronomy* (Agronomic Affairs, 1923b), p. 528

These registered cereals became the standards for evaluating whether subsequent applications gained scientific distinction as a variety. An involution in pedigree followed. That standard varieties increasingly served as parentage in the breeding of new varieties made direct comparison easier. Any differences in acre-yield (bushels) were readily documented by side-by-side comparison with a prior registered variety.^24^ Plus, breeding history disclosed the likely source of whatever production gains the newer variety exhibited (Fig. 4).

The amount of work in registering the initial three cereals suggested that the task could overwhelm the Committee. The members made a decision to further limit eligibility. “Only varieties or strains of known breeding history” would be accepted for registration (Agronomic Affairs, 1927). The refusal to consider crops of unknown origin redefined breeding value explicitly in terms of pedigree and yield. Registration of other commodity crops followed this model. Consideration of open-pollinated corn met with categorical rejection in 1927 (Agronomic Affairs, 1927); whereas two years later, the Committee welcomed registration for inbred strains of corn (Agronomic Affairs, 1929). Likewise, in response to a request for cotton registration, Committee members replied that “a system of controlled self pollination” was needed before cotton varieties exhibited the “practical homozygosity” required for registration (Agronomic Affairs, 1928). The proliferation of requests called for checks on the administrative body and its workload. The Committee revised its format in 1929, dispensing with the special subcommittees for each crop (Agronomic Affairs, 1930). The per-crop organizational structure could be abandoned because, by that point, general protocols for adducing varietal identity were in place.

The classification, testing, and registration of named crop varieties ensured against duplication by guaranteeing a unique denomination for any entry with an known pedigree and superior yield.^25^ Although registration granted no legal protection, the varietal review established by the Committee would have implications for later intellectual property legislation (Ellis et al., 2010). In part, this is because an ability to identify what is being protected is a basic requirement of intellectual property. Other means of dealing with varietal identification, outside the scope of the federal registry, would continue for vegetable crops.

## Don’t forget to cite your vegetables

What I have shown is that, prior to 1930, the Committee skirted questions about the ontological standing of crop varieties through an innovative reworking of the defining properties of a “variety.” Registration was one method of inscribing surety and discouraging synonymy. Yet only major commodity crops received early consideration for registration. The amount of work involved proved impracticable for the volunteer labor of the Committee. Vegetables comprised a separate category than cereals and, as such, varietal standardization for vegetables proceeded outside the American Society of Agronomy registry. How did scientists and seedsmen present a unified version of a crop variety for public knowledge without resorting to a federal depository? Without a material sample to substantiate the varietal description, the words lacked flesh, to reinvoke Daston’s memorable phrase.

In 1929, the USDA Division of Horticultural Crops and Diseases began to publish a description of important vegetable varieties—starting with tomatoes, cabbages and garden peas (Boswell, 1932). Earlier efforts to set varietal standards for these same crops seem to have been inconsequential, largely due to interruptions from World War I (McCue, 1919, p. 71) This new federal undertaking was, as before, based on widespread confusion surrounding the concept of a variety. Ambiguity beset all manner of crops in the “twilight zone” below the species rank, and the same explanation was given across homologous institutional settings doing similar kinds of work. Common protocols were needed for the purposes of making a more positive identification.

Akin to the initial survey work of cereal registration, a total of nineteen state experiment stations contributed to the USDA project for standardizing vegetables. Each participating site agreed on a set of standards for recording the appearance and performance of each variety. A published description and color illustration gave confidence to varietal names, capturing the “unavoidable deviations from the standard type” in different regions across the United States (Boswell, 1934). The descriptions of each varietal type were admittedly “somewhat idealistic” rather than a full appraisal of extant offerings (Magruder et al., 1940). Still, the illustrations served a similar purpose as the material deposit used for cereals insofar as the images substantiated the written description. Through the system of varietal descriptions and type samples, researchers familiar with each crop began to reach an agreement, while shying away from retail catalogue copywriting. One benefit of the project, justifying its undertaking, was a “reduction in number of varieties” (Magruder et al., 1941). Crop scientists apparently found more provincial labels in the course of their work than bona fide crop varieties.

Again, economic priority ruled. The publications covered high-value crops, not miscellaneous vegetables like pimento peppers.^26^ Returning to pimento provides a case in point for understanding the history of varietal standardization. Shifty seedstocks could, over time, ruin a seed firm’s reputation, but varietal flux caused greater immediate financial injury to growers on the farm.

### “Wouldn’t you like to be a pimento, too?”

**Fig. 5:**
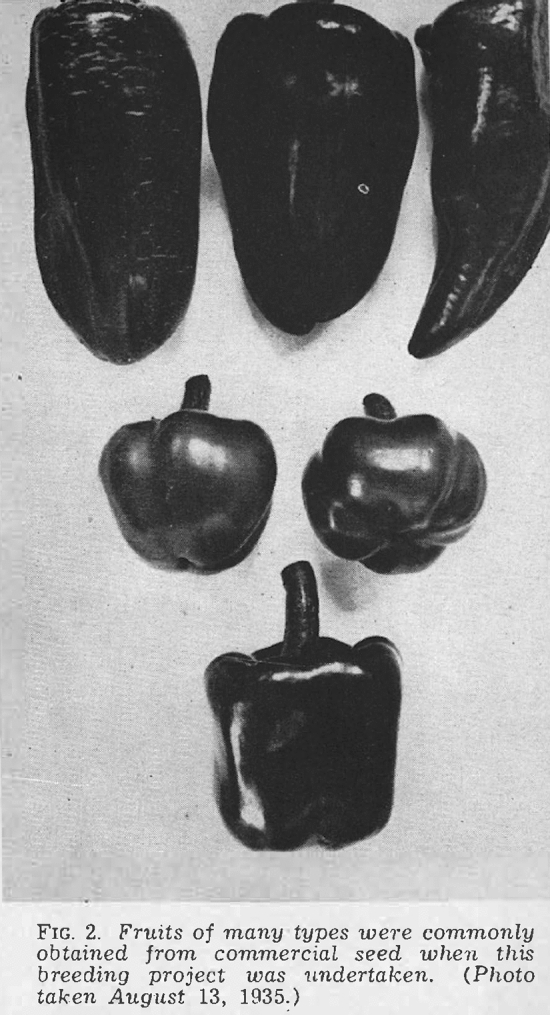
Variability in Perfection peppers. Reproduced from Cochran (1943), p. 5

As detailed, the entire Georgia pimento crop descended from seed selected by Riegel and Sons in Experiment, Georgia. Perfection was not the same as Pimento; it was a variety of pimento and the best in the business. Until it wasn’t. By the 1930s, “growers and canners described [Perfection] as having ‘run out’ because the commercial seed produced fruits of many shapes and sizes” (Cochran, 1943, p. 3). Pimento growers in middle Georgia suffered from too much variability within the popular variety (see Fig. 5). This was not a matter of synonymy, or extra varieties going under the same name. Instead, Perfection no longer bred true to its original type when planted from seed. The overall pimento acreage in Georgia underwent a drastic decline due to varietal instability. Canners refused to pack diminutive or irregular peppers, and yields became unprofitable.

Agronomists at public breeding institutions took notice of variation within Georgia pimento fields. Other breeders also took notice and seized it to their advantage. Nora Jenkins wrote to the Georgia Experiment Station in 1931 requesting a bulletin on growing pimentos.^27^ In the letter, she described herself as a “breeder of a very fine strain.”^28^ Director Stuckey promptly asked her for a sample to trial against the station’s own selections. In her reply, Jenkins reiterated the “unquestionable” purity of her strain, having developed it from a single precocious plant and in complete isolation from other peppers, selecting for thick fruit of uniform type over a thirteen-year period. This method of selection, known as pedigree breeding, was a popular practice among commercial breeders and public scientists. Testifying to the distinction of her crop, Jenkins wrote that other people “passing through the pimento fields of Georgia claim mine to be far superior.” She was sure to add, “you understand, of course, that I sow and control the entire strain.”^29^ This ownership claim, along with the reputation gained for her ongoing work, was familiar to the old moral economy of breeders (e.g. Charnley, 2013b). What proprietary feelings Jenkins held over her pimentos did not prohibit her from sending seed to Stuckey. Plant geneticists in the United States may have unofficially denied amateur breeders involvement in scientific crop improvement, but their seed was welcome all the same. 

The Georgia Experiment Station began its own pedigree breeding in 1935. The objective was to select for a higher percentage of top-grade, uniform, heart-shaped Perfection stock. Much like Jenkins, their new variety started from a “single plant selection of the Perfection Pimento in a farmer field”—only this time, it was further inbred beneath muslin cages (Cochran, 1943). After seven years of pedigree breeding, the Georgia Experiment Station published a bulletin at the release of “Truhart Perfection.” Truhart was a kind of Perfection, just as Perfection was a kind of pimento, which itself was a kind of *C. annum*. The new variety possessed the thick fruit walls and carmine red color desired for packing pimentos in glass jars (Cochran, 1943). The Georgia Experiment Station shared foundational seed directly with canners in Georgia. It proved to be the most popular pimento variety through the 1960s (Brackett, 2018).

### Standardized forms and plant intellectual property

Soon after the release of Truhart, the pimento breeding program at Georgia relocated to the neighboring state of Alabama, where the Auburn Experiment Station released the “Bighart” variety in 1969 (Greenleaf et al., 1969). Bighart embodied an historical change in breeding practice and crop biology. Again the new pimento variety originated from a chance plant with extra-large fruit. Yet rather than isolate its offspring, as was typical in the first half of the twentieth century, the Bighart pedigree included Truhart Perfection and five other varieties, developed over 36 generations. Bighart was not a questionable synonym of Truhart or another refinement of Reigel’s claim to fame. Extensive crossing with a wide array of *C. annum* differentiated this pepper from all other pimentos, even absent registration.

The following year, passage of the 1970 *Plant Variety Protection Act* in the United States extended intellectual property rights to sexually-reproduced plants.^30^ Applicants for plant variety protection were required to submit a material sample along with descriptive data to distinguish the variety from comparable accessions. The distinct, uniform, and stable criteria of the 1970 *Plant Variety Protection Act* provided the techno-scientific means for arbitrating whether or not a crop variety was eligible for intellectual property protection. (Under the law, an added criterion of "novelty" decided questions of prior existence within a jurisdiction.) A notable similarity, therefore, exists between the legal requirements of plant variety protection and the stipulations established for registration earlier in the century.

By 1970, the system of crop registration covered varieties (cultivars) and breeding lines for many crop species (Ellis et al. 2010). Brad Sherman (2008) has argued that taxonomy resolved questions of botanical identity through the assignment of a unique name, readying plants for review under intellectual property law (576). The history of varietal standardization in the United States, however, demonstrates that general systematics ceased to apply below the species rank. Questions of quiddity among agricultural crops departed from the classification of botanical taxa in systematics. Crop scientists adopted a different set of criteria for registration. That this history largely occurred before legal regimes formalized intellectual property in plants indicates why the epistemic concept of a “variety” is worthy of further analysis, in the United States and beyond.^31^

What I have shown is that crop scientists were still figuring out how to conceptualize a variety during the first few decades of the twentieth century. At first, agronomists in the United States sought to identify varieties with morphological characteristics, the way botanists did, but this taxonomic approach was useless for breeding purposes. Another method of identification was required. This objective was partially resolved through the defining properties of a variety, especially with regards to hybrid breeding. While agreement on a singular name was a matter of compulsory grammar rules and publication rites, it was the recourse to breeding history and comparison with existing standards that validated claims to crop varietal distinction. In this way, standardization of crop varieties in the first half of the twentieth century carried over into intellectual property through a shared set of administrative criteria.

To conclude, neither a reliable designation nor biological stability would control the protectability of pimentos. A powerful food manufacturer was opposed to it. The Campbell Soup Company successfully lobbied for the 1970 *Plant Variety Protection Act* to exclude six vegetables, including peppers.^32^ Concerns about economic viability, once again, outranked other criteria in debates over crop varieties.

## Footnotes

1 Stuckey to Hastings, 2 January 1930, University Archives (UA), Georgia Experiment Station Materials (1879–1999), H.P. Stuckey: Correspondences, 07–022, Box 9, Folder 1.

2 Spelling of pimento varies in print, as some people used the term “pimiento”.

3 Hastings to Stuckey, 3 January 1930, UA 07–022, Box 9, Folder 1.

4 The origin of this descriptive phrase is beyond my knowledge, but, as one reviewer noted, deserves historical scrutiny in its own right.

5 Stuckey to Wood, 2 April 1930, UA 07–022, Box 9, Folder 2.

6 Diversity within *C. annuum* has baffled experts. In fact, the difficulty of classifying within the species led American geneticists to propose the commercial bell-pepper variety “California Wonder” as the type specimen of the entire species, including its wild progenitors! See Csilléry (2006), p. 153.

7 A year prior, the Iowa State Agricultural Experiment Station published a bulletin on home vegetable gardens that contained a list of dependable varieties, with mention of Pimento but not Perfection (Haber, 1929).

8 Stuckey to Wood, 28 March 1930, UA 07–022, Box 9, Folder 2.

9 Stuckey to Hastings, 2 January 1930, UA 07–022, Box 9, Folder 1.

10 *Hastings’ Seeds*. Cherokee Gardens Library, Kenan Research Center, Atlanta History Center, Hastings’ Seed Company Records MSS 992, Box 2, Folder 12.

11 Hastings to Erwin, 3 January 1930. UA 07–022, Box 9, Folder 1.

12 Ibid.

13 Erwin to Hastings, 9 January 1930, UA 07–022, Box 9, Folder 1.

14 This history was recounted by Ellis et al. (2010), and I owe inspiration for my review of these archives to their original work.

15 That each language contains a plant name is not admitted as synonymy, being covered by “translation”, yet the two concepts share the problematic of understanding what is being signified.

16 Bert Theunissen (2011) has argued the inverse, that Darwin came to understand pigeon breeding through his theory of natural selection. In either case, the difference is irrespective of rank.

17 Dominic Berry (2019) provides a thorough review of this massive body of literature.

18 As quoted in the opening of the article, Harlan (1957) reflects on this with great hilarity.

19 The term “horticulture” encompassed various garden activities, including fruit and flower breeding, but generally not agricultural field crops.

20 In a separate context, Javier Lezaun (2012) has argued that legal reference materials impose a “relative inertness” to the flux of physical object, conveying the technical means for displacing original source material with an authoritative version.

21 Here, crop refers to an annual plant sown by seed. Trees and perennials are not included.

22 Hastings to Erwin, 3 January 1930, UA 07–022, Box 9, Folder 1.

23 Of 138 cases prosecuted by BPI from 1931–1937, I found that only 21 involved a contest over varietal identity.

24 The exact parameters employed by government stations to measure varietal performance is beyond the scope of this article. See special issue of *HPLS* with Guiditta Parolini (2015) for more.

25 Naming of varieties, or cultivars, would be governed by conventions in the International Code of Nomenclature for Cultivated Plants. Not all varieties registered by the Committee abided the rules of the Code and a separate committee was recommended in 1949 to revisit the problem (Agronomic Affairs, 1950).

26 The USDA bulletins included the principal varieties of spinach, orange-fleshed carrots, red beets, and onions before World War II put an end to the project.

27 See Stuckey & McClintock (1921).

28 Jenkins to Stuckey, 5 March 1931, UA 07–022, Box 9, Folder 18.

29 Jenkins to Stuckey, 11 March 1931, UA 07–022, Box 9, Folder 18.

30 Public Law 91–577 (24 Dec. 1970) 84 Stat. 1542–1559.

31 The U.S. *Plant Patent Act* of 1930 was the first of its kind anywhere in the world to include plant varieties within intellectual property law, but the scope of the law was limited to asexually-reproduced plants. See Fowler (2000).

32 The stated reason was a concern for rising soup prices. See Bugos and Kevles (1992), p. 94. The U.S. lifted the exemption on these soup crops for international legal harmonization in 1980.
